# PhenoExam: gene set analyses through integration of different phenotype databases

**DOI:** 10.1186/s12859-022-05122-x

**Published:** 2022-12-31

**Authors:** Alejandro Cisterna, Aurora González-Vidal, Daniel Ruiz, Jordi Ortiz, Alicia Gómez-Pascual, Zhongbo Chen, Mike Nalls, Faraz Faghri, John Hardy, Irene Díez, Paolo Maietta, Sara Álvarez, Mina Ryten, Juan A. Botía

**Affiliations:** 1 Departamento de Ingeniería de la Información y las Comunicaciones, Universidad de Murcia, Murcia, Spain; 2 Department of Neurodegenerative Disease, UCL, Institute of Neurology, London, UK; 3 Data Tecnica International LLC, Glen Echo, MD USA; 4 Laboratory of Neurogenetics, NIA/NIH, Bethesda, MD USA; 5 Center for Alzheimer’s and Related Dememtias, NIH, Bethesda, MD USA; 6 NIMGenetics Genómica y Medicina S.L, Madrid, Spain; 7 NIHR Great Ormond Street Hospital Biomedical Research Centre, University College London, London, UK; 8 Genetics and Genomic Medicine, Great Ormond Street Institute of Child Health, University College London, London, WC1E 6BT UK; 9 Reta Lila Weston Institute, UCL Queen Square Institute of Neurology, London, UK; 10 UCL Movement Disorders Centre, University College London, London, UK; 11 Institute for Advanced Study, The Hong Kong University of Science and Technology, Hong Kong, China

**Keywords:** Phenotype Enrichment Tool, Shiny, Epilepsy

## Abstract

**Background:**

Gene set enrichment analysis (detecting phenotypic terms that emerge as significant in a set of genes) plays an important role in bioinformatics focused on diseases of genetic basis. To facilitate phenotype-oriented gene set analysis, we developed PhenoExam, a freely available R package for tool developers and a web interface for users, which performs: (1) phenotype and disease enrichment analysis on a gene set; (2) measures statistically significant phenotype similarities between gene sets and (3) detects significant differential phenotypes or disease terms across different databases.

**Results:**

PhenoExam generates sensitive and accurate phenotype enrichment analyses. It is also effective in segregating gene sets or Mendelian diseases with very similar phenotypes. We tested the tool with two similar diseases (Parkinson and dystonia), to show phenotype-level similarities but also potentially interesting differences. Moreover, we used PhenoExam to validate computationally predicted new genes potentially associated with epilepsy.

**Conclusions:**

We developed PhenoExam, a freely available R package and Web application, which performs phenotype enrichment and disease enrichment analysis on gene set G, measures statistically significant phenotype similarities between pairs of gene sets G and G′ and detects statistically significant exclusive phenotypes or disease terms, across different databases. We proved with simulations and real cases that it is useful to distinguish between gene sets or diseases with very similar phenotypes.

Github R package URL is https://github.com/alexcis95/PhenoExam.

Shiny App URL is https://alejandrocisterna.shinyapps.io/phenoexamweb/.

**Supplementary Information:**

The online version contains supplementary material available at 10.1186/s12859-022-05122-x.

## Background

One of the main aims of clinical genetics research is to discover new gene-disease associations [1–6]. A disease is commonly diagnosed through the identification of a set of symptoms and signs associated with a particular and recognized clinical phenotype [7–10]. While some phenotypes are due to the impact of environmental factors, if a disease is inherited then the genetic variation within the individual also explains the phenotype at least partially [11]. Here, we introduce PhenoExam, a software tool to assist in the identification of new gene-phenotype associations. PhenoExam focuses on genetic diseases, harnessing all available gene-phenotype annotation resources to provide a comprehensive gene set and differential gene set annotation approach.

Over the last decade, we have seen attempts to standardize our knowledge of genetic diseases by formally linking genes to phenotypes using standard terminology, as exemplified by The Human Phenotype Ontology (HPO) [12] and The Mouse Genome Database (MGD) [13]. HPO is a standardized set of human phenotypic terms that are organized hierarchically with a directed acyclic graph and have been used to annotate all clinical entries in the Online Mendelian Inheritance in Man database (OMIM). OMIM [14] is a continuously updated catalog of human genes, genetic diseases, and traits, with a particular focus on the molecular relationship between genetic and phenotypic variation. On the other hand, MGD is the manually curated consensus representation of genotype to phenotype information including detailed information about genes and gene products. It is the authoritative source for biological reference data sets related to mouse genes, gene functions, phenotypes, and mouse models of human disease. MGD has more terms and detailed phenotypic information than HPO because scientists can perform a wider set of experiments on mice. These features increase our knowledge and can help to prioritize novel gene-phenotype relationships in humans. Beyond phenotype databases, PhenoExam also includes gene-disease association databases, namely UniProt [15], The Comparative Toxicogenomics Database (CTD) [16], Orphanet [17], The Clinical Genome Resource (ClinGen) [18], The Genomics England PanelApp [19], The Cancer Genome Interpreter (CGI) [20] and PsyGeNET [21]. It also includes CRISPRbrain [22], the first genome wide CRISPR interference and CRISPR activation screen in human neurons so we may study the potential association of phenotypic terms to specific functions of these genes in human neurons.

Apart from being a general-purpose tool for phenotype-based gene sets annotation, PhenoExam can also help in the diagnosis of genetic diseases. Currently fewer than half of patients with suspected Mendelian disorders (genetic diseases primarily resulting due to alterations in one gene) receive a molecular diagnosis [23]. Diseases with a genetic basis are usually diagnosed by looking for causal mutations in a panel of genes specifically associated with the disease. Gathering all phenotypes associated with the genes in a panel delivers a general phenotype-level description beyond the disease under study. To improve the accuracy of genetic diagnosis, we need methods to appropriately evaluate the gene level phenotypic similarity between candidate diseases. Moreover, the identification of differential phenotypes between diseases can also help towards more precise diagnostics. The identification of exclusive and/or shared phenotypes between gene panels can demonstrate common pathophysiology [24] but it can also help to create genetic links between diseases through their gene sets [25, 26]. We can find numerous methods based on measuring disease-based phenotypic similarities by comparing sets of HPO terms e.g., Phenomizer [27], HPOSim [28], and PhenoSimWeb [29], Table 1 offers a detailed comparison amongst all tools. We also have modPhEA [30], an online resource for phenotype enrichment analysis. modPheEA helps with the gene-based phenotype enrichment analysis but just focused on one phenotype database at a time and without considering conditional analyses (two gene sets).

**Table 1:** Comparison of PhenoExam and other similar tools. “X” means the tool provides the function and “–” means the tool does not. “*” means the similarity scores are between phenotype terms and not between gene sets as does PhenoExam

Tool	As web	As software tool	Open source	Model Organism	Phenotype sets	Gene sets	Multiple database at once	Phenotype Enrichment Analysis	Disease Enrichment Analysis	Differential phenotypes	Diagnosis based on phenotypes	Similarity scores
PhenoExam	X	X	X	X	X	X	X	X	X	X	–	X
modPhEA	X	–	–	X	X	X	–	X	–	X	–	–
DisGeNET	X	X	X	–	X	X	X	–	X	–	–	–
Phenomizer	X	–	–	–	X	–	–	–	–	–	X	*
HPOSim	–	X	X	–	X	X	–	X	–	–	–	*
PhenoSimWeb	X	–	–	–	X	X	–	–	–	–	–	*

Phenomizer obtains the phenotype semantic similarity between sets of phenotypes based on the HPO ontology but does not rely on the use of the genes implicated in each phenotype. HPOSim is an R package that implements widely used ontology-based semantic similarity measurements to quantify phenotype similarities, and phenotype-level enrichment analysis using a hypergeometric test and the NOA method [31]. PhenoSimWeb is an online tool for measuring and visualizing phenotype similarities using HPO, uses a path-constrained Information Content-based measurement in three steps and exploits the PageRank algorithm [32]. Nevertheless, these tools did not take some important concepts into consideration. PhenoExam contributes to the field with new features. These include the ability to detect differential phenotypes between pairs of gene sets: phenotypes that are significant within one gene set only, useful for detecting featured phenotypic terms between gene sets to distinguish better between similar diseases. It also combines phenotype and disease terms. This is important to link phenotypes to specific diseases. Finally, it tries to make the interpretation of the results of the phenotypic analysis easier by using simple scores to rank significant terms as well as summary messages and interactive graphs. We also found a knowledge management platform integrating and standardizing data about disease-associated genes from multiple sources called DisGeNET [33]. While being similar to PhenoExam in finding gene-disease associations, DisGeNET does not, however, offer facilities for gene-based phenotype enrichment analysis or for detecting phenotypic conditional similarities between pairs of gene sets. PhenoExam uses as the basic substrate for gene-phenotype and gene-disease associations a number of configurable databases both in human and mouse that the user can tailor and adapt depending on the type of analysis to be performed. In PhenoExam, the phenotypic similarity between two groups of genes is performed by assessing the statistical significance of the Phenotypic Overlap Ratio (POR) between those (i.e., the number of common enriched phenotypes between the gene sets) (See methods Phenotype scores calculation).

We developed PhenoExam intending to support a variety of target users, mainly clinicians, computational biologists, and geneticists. PhenoExam can help clinicians with finding phenotypes which are exclusive to diseases amongst a set of possible genetic disease candidates whose diagnosis is based on gene sequencing panels (Case 1). PhenoExam is also useful for geneticists as it can be used to improve their in-house-maintained gene panels but also to more accurately select genes involved in specific genetic studies (Case 2). Finally, computational biologists can use PhenoExam to discover new information about gene sets of interest thanks to the integration of multiple phenotype and disease databases and to compare phenotypes between known genes associated with a disease and the validation of computationally predicted disease genes (Case 2).

## Design and implementation

### Database integration

**Fig. 1:**
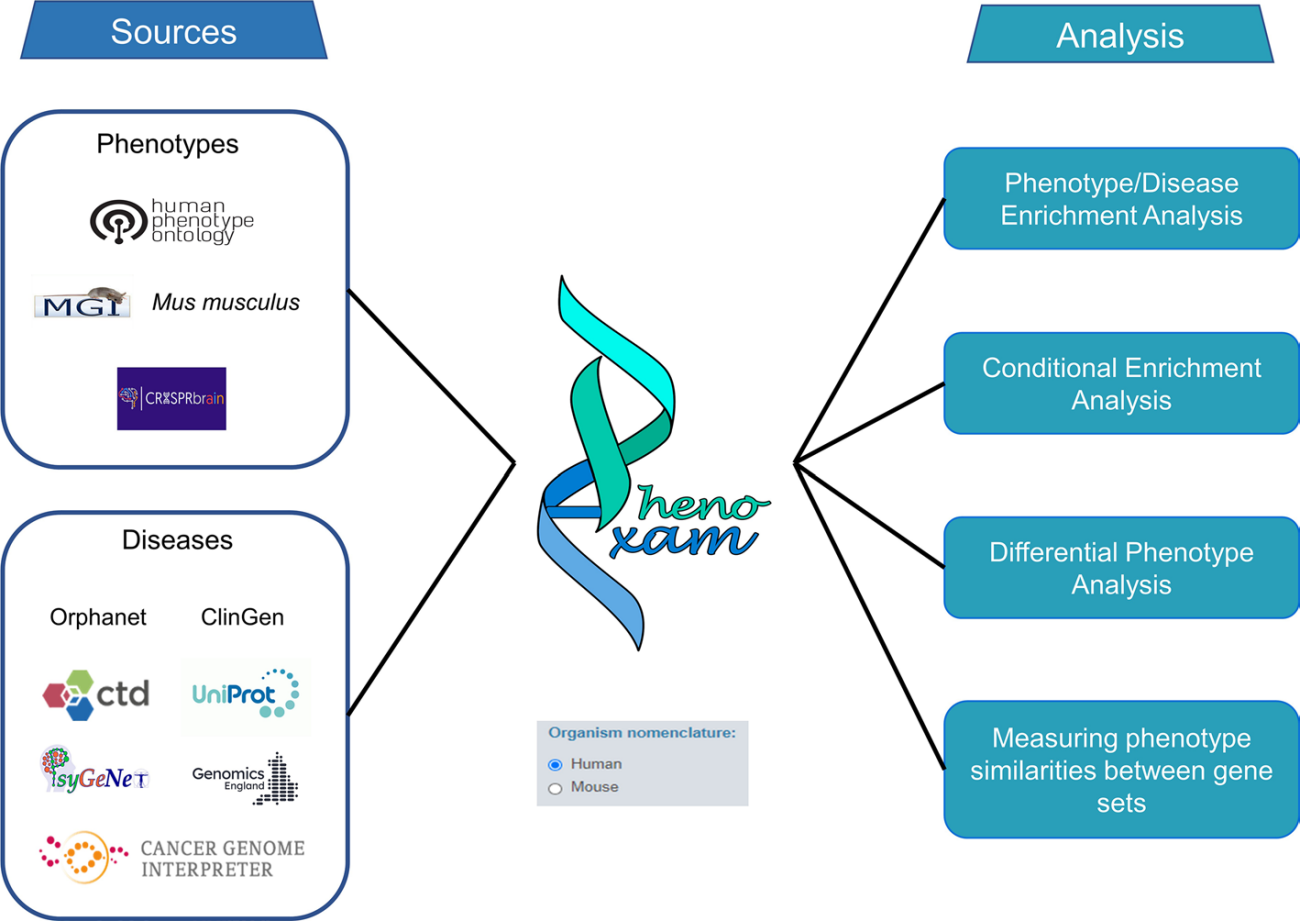
Schematic representation of PhenoExam integrated databases and offered analyses. We can use PhenoExam with human or mouse genes. PhenoExam annotation databases include HPO, MGI, CRISPRBrain, CTD, ClinGen, OrphaNET, UniProt PsyGeNET, CGI and Genomics England. The tool offers a variety of analyses. Given a gene set of interest, G, the user can evaluate its enrichment for phenotypes and disease in all or a subset of the offered databases. Given two gene sets, G and G′, the user can evaluate whether the phenotype terms enriched in G are also enriched in G′ when G and G′ do not overlap e.g., G′ was predicted from G, with the Conditional Enrichment Analysis. If G and G′ show some gene overlap, the user can assess whether the gene sets show any differential phenotypes through the Differential Phenotype Analysis. We acknowledge all the sources for their contributions and we are grateful to those who permitted us to use their logos in this figure

The set of analyses performed by PhenoExam is based on manually curated phenotypes language like HPO, gene-disease ones as OMIM but also screening-based databases like CRISPRBrain, amongst many others (see Table 2 for a complete list, description, and potential use). PhenoExam can perform a variety of analyses (Fig. 1). The integration of these different databases is possible thanks to a well-established standardization process of genes and phenotypes used by PhenoExam. Using the HUGO Gene Nomenclature Committee (HGNC) gene naming system as the common way of identifying all human genes, and the definition of a new annotation term within each annotation database to indicate the HGNC genes that do not have any phenotype term associated in the database of interest. The list of HGNC genes was obtained from [34] https://www.genenames.org/download/statistics-and-files/. The HPO gene-phenotype association list was obtained from https://archive.monarchinitiative.org/latest/tsv/gene_associations/. The new no-phenotype association (HPO:XXX No HPO phenotype) was added to HPO for all protein coding genes with no known association to phenotype. For MGD, MP terms from orthologous genes to humans were obtained from http://www.informatics.jax.org/downloads/reports/index.html#go, and the relationship between human genes—mouse genes—mouse phenotype were collected using the files (MGI_PhenoGenoMP.rpt, HMD_HumanPhenotype.rpt, VOC_MammalianPhenotype.rpt). A new no- phenotype association (MP:XXX No phenotype) was created and all the protein coding genes without a relation to phenotype were linked to this term. For CRISPRBrain, the gene-phenotype relationships were obtained from https://crisprbrain.org/simple-screen/. For the generation of this database, the phenotypes were codified in three classes for each CRISPR analysis: association to the phenotype (Positive-Hit and Negative-Hit genes in CRISPRBrain), positive association (Positive-Hit genes in CRISPRBrain) and negative association (Negative-Hit genes in CRISPRBrain). This was accomplished according to the Hit-Class label in CRISPRbrain (Positive-Hit, Negative-Hit). The non-relationship phenotype (CRB:XXX No phenotype was created and all the protein coding genes that were not related to any phenotype were related to this term. We integrate into PhenoExam only the information from curated databases (UniProt, CTD, Orphanet, ClinGen, The Genomics England PanelApp, CGI and PsyGeNET). Then the non-relationship disease term (CXXX No diseases associated) was created and all the protein coding genes that were not related to any disease were related to this term. After standardization process, the current release (v1.0) of PhenoExam contains, 659,634 gene-phenotype associations, involving 20,209 genes, 18,159 different phenotypes and 9348 different diseases (see details in Table 2).

**Table 2:** Databases usable through PhenoExam and size of each in terms of genes, phenotypes and associations. Numbers reported are final, after preprocessing and unification of gene names across databases

Source	Genes	Phenotypes	Diseases	Assocs	Summary
HGCN	19,197	–	–	–	All protein coding genes
HPO	19,248	7861	–	186,290	Human gene-phenotype associations
MGD	17,900	10,243	–	242,313	Mouse gene-phenotype associations
CRISPRBrain	19,275	55	–	43,481	Cell screen gene-phenotype associations
ClinGen	19,198	–	420	19,851	Human gene-disease associations
Genomics England	19,230	–	5538	24,336	Human gene-disease associations
CTD	19,636	–	6843	58,660	Human gene-disease associations
CGI	19,198	–	177	20,361	Human gene-disease (cancer) associations
UniProt	19,204	–	3868	21,101	Human gene-disease associations
Orphanet	19,262	–	3183	2228	Human gene-disease (rare) associations
PsyGeNET	19,248	–	82	20,952	Human gene-disease associations
ALL	20,209	18,159	9348	544,022	PhenoExam tool

### Phenotype scores calculation

#### Phenotype enrichment analysis on a gene set G

PhenoExam obtains a list of statistically significant enriched phenotypes in a given set of gene *G* within a phenotype/disease database annotation of reference D. In order to calculate whether a gene set *G* shows enrichment in a given phenotypic term *p* belonging to D, let g be the number of genes in G associated with p. Let also gdb be the number of genes associated with p and GDB the total number of genes in the database, we model the enrichment probability with a hypergeometric distribution such that:

$$P\left(X=g\right)=\frac{\left(\genfrac{}{}{0pt}{}{gdb}{g}\right)\left(\begin{array}{ccc}GDB& -& gdb\\ \left|G\right|& -& g\end{array}\right)}{\left(\genfrac{}{}{0pt}{}{GDB}{\left|G\right|}\right)}$$

Any phenotype with *P* < 0.05 will be enriched in the *G* gene set. We compute this probability for each phenotypic term *ph* associated with 1 gene or more in *G* and use these probabilities as *P* values. PhenoExamWeb reports the raw, Bonferroni [35] and false discovery rate (FDR) [36] adjusted *P* values.

#### Phenotypic Overlap Ratio score

PhenoExam´s approach to measuring the similarity between two gene sets G and G´, within an annotation database D, is based on a score called the Phenotypic Overlap Ratio (POR). Let Gp be the number of significantly enriched terms in D for genes in G, and analogously for G′p. The POR could be computed using the widely used Jaccard index or the Forbes similarity coefficient corrected by Alroy [37] on the agreement between the subsets of significant phenotypes. PhenoExam allows users to choose between these two options accordingly to Salvatore et al. [38]*.* conclusions.

Jaccard index:

$$POR\left(G,{G}^{{\prime}}\right)=\frac{Gp \cap G{^{\prime}}p}{Gp \cup G{^{\prime}}p}$$

Forbes similarity coefficient corrected by Alroy:

$$N=Gp \cap {G}^{{\prime}}p + Gp {\setminus} {G}^{{\prime}}p + {G}^{{\prime}}p {\setminus} Gp$$

$$POR\left(G,{G}^{{\prime}}\right)=\frac{Gp \cap {G}^{{\prime}}p*(N+\sqrt{N})}{[\left(Gp \cap {G}^{{\prime}}p+ Gp {\setminus} {G}^{{\prime}}p\right)*\left(Gp \cap {G}^{{\prime}}p + {G}^{{\prime}}p {\setminus} Gp\right)+Gp \cap {G}^{{\prime}}p*\sqrt{N}+(Gp {\setminus} {G}^{{\prime}}p* {G}^{{\prime}}p {\setminus} Gp)/2]}$$

POR (*G, G*′) takes values in [0,1], resulting in 0 when no phenotype is shared and 1 when the sets share all phenotypes (Jaccard index) or at least share all phenotypes from one set (Forbes coefficient).

#### Statistically significant Phenotypic Overlap Ratio

PhenoExam assess whether the POR between gene sets G and G′ is statistically significant by means of randomization. We will have two modalities of the POR, depending on whether G and G′ share genes or, on the contrary, they are disjunct (e.g., G′ was predicted from G). When G and G′ are thought to share genes, POR (*G, G*′) is compared with POR (*G, R*) and with POR (*G*′*, R*′), where *R* has the same size as G and R′ the same as G′. Genes in both R and R′ are chosen randomly within the whole set of protein coding genes. We repeat this process for m random gene sets $({R}_{1},{R}_{2},\ldots,{R}_{m})$ and $\left({{R}^{{\prime}}}_{1},{{R}^{{\prime}}}_{2},\ldots,{{R}^{{\prime}}}_{m}\right)$ to obtain an empirical *P* value with the proportion of random gene sets whose POR is greater than the observed one. On the other hand, when G′ is obtained by using G as input of the generation process, we say G′ is conditioned to G. Therefore, the significance test of the POR (G, G′) is reduced now to obtain an empirical *P* value based on the proportion of times a randomized POR (G, R), with R any of $({R}_{1},{R}_{2},\ldots,{R}_{m})$ all with the same size of G while keeping G constant, shows higher values than the observed POR (G, G′).

#### Relaxed Phenotypic Overlap Ratio

The POR only considers phenotypes that were assessed as statistically significant. Sometimes, it may be of interest to relax this restriction to incorporate all phenotype/disease terms associated with G. In this case, the score is called Relaxed Phenotypic Overlap Ratio (RPOR). It is calculated in a similar way to the POR but with all phenotypes, whether these are enriched or not. In the same way, as with the POR, we can determine whether the RPOR is statistically significant by using randomization.

#### Phenotype relevance association analysis for gene sets

Once it has been determined that two sets of G and G' genes share some enrichment of phenotypic terms, and focusing only on the shared terms, we can measure the correlation of the number of genes of each phenotypic term as measured in G and G′ by a linear regression model and report the R^2^ as the strength of this correlation together with the association *P* value. Higher values of R^2^ would suggest a linear association between importance of phenotypic terms in G and importance of the same genes in G′.

### Generation of the web interface

We have developed PhenoExamWeb, a web-based tool for performing phenotypic analyses using R. PhenoExamWeb shiny app is accessible at https://alejandrocisterna.shinyapps.io/phenoexamweb/. R and the shiny R package [39] were used for front-end scripting of the web interface. R scripts were used for back-end execution and analysis with the development environment of R version 3.6.3. The R package is available at https://github.com/alexcis95/PhenoExam. Note that although we offer PhenoExam through a Web application, it might be a better option to consider installing and using the R package locally for the sake of flexibility or to deploy the shiny app locally in your local workstation for computationally demanding analyses like, for example, a “comparator phenotype analysis” with more than 40 random tests. Simply download the software from https://github.com/alexcis95/PhenoExam/blob/master/PhenoExamWeb.zip and run the Rmd file locally.

### Analysis with PhenoExamWeb

**Fig. 2:**
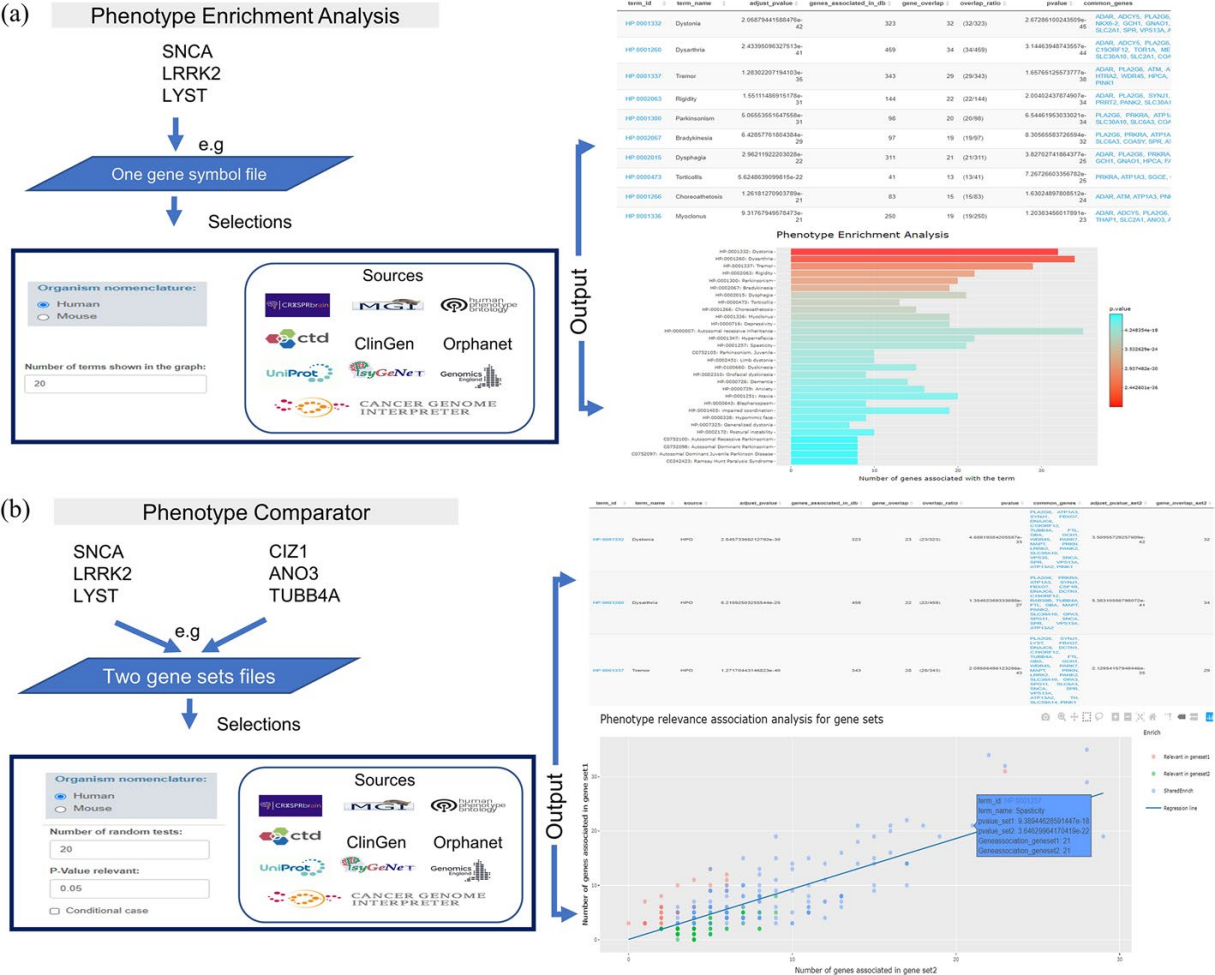
PhenoExamWeb shiny app possible workflows. **a** Phenotype Enrichment Analysis: requires one gene symbol file as input file, which gene symbol nomenclature (Organism nomenclature: Human or Mouse) we use, the phenotype/disease annotation databases to be considered and the top number of terms shown in the graph. The results generate an interactive table and graph which include phenotypes, genes implicated with each term and *P* values as output. **b** The Phenotype Comparator requires two gene sets as input together with the gene symbol nomenclature (Human or Mouse) used, the annotation databases of interest for the analysis and the number of random tests to obtain empirical *P* values, the relevant *P* value threshold and whether our analysis is a conditional case (i.e., if one gene set was generated after a prediction analysis from the other and they are totally different gene sets). Finally, we obtain the summary of the analysis with the similarities phenotype scores, the differential phenotypes, interactive tables and graphs with phenotypes, genes and *P* values as output for detailed inspection and result presentation. We acknowledge all the sources for their contributions and we are grateful to those who permitted us to use their logos in this figure

PhenoExamWeb requires gene symbols, human or mouse, as the input file. Then, we need to select the type of analysis: Phenotype Enrichment Analysis (One gene set) or Phenotype Comparator (Two gene sets). We also need to specify the database or databases. The workflow of PhenoExamWeb is summarized in Fig. 2. Users can follow the web tutorial on the website (https://alejandrocisterna.shinyapps.io/phenoexamweb/#section-help) and the R package tutorial on GitHub (https://raw.githack.com/alexcis95/PhenoExamWebTutorials/main/tutorial.html).

## Results and discussion

### PhenoExam controls type I error when used with all phenotype databases

**Fig. 3:**
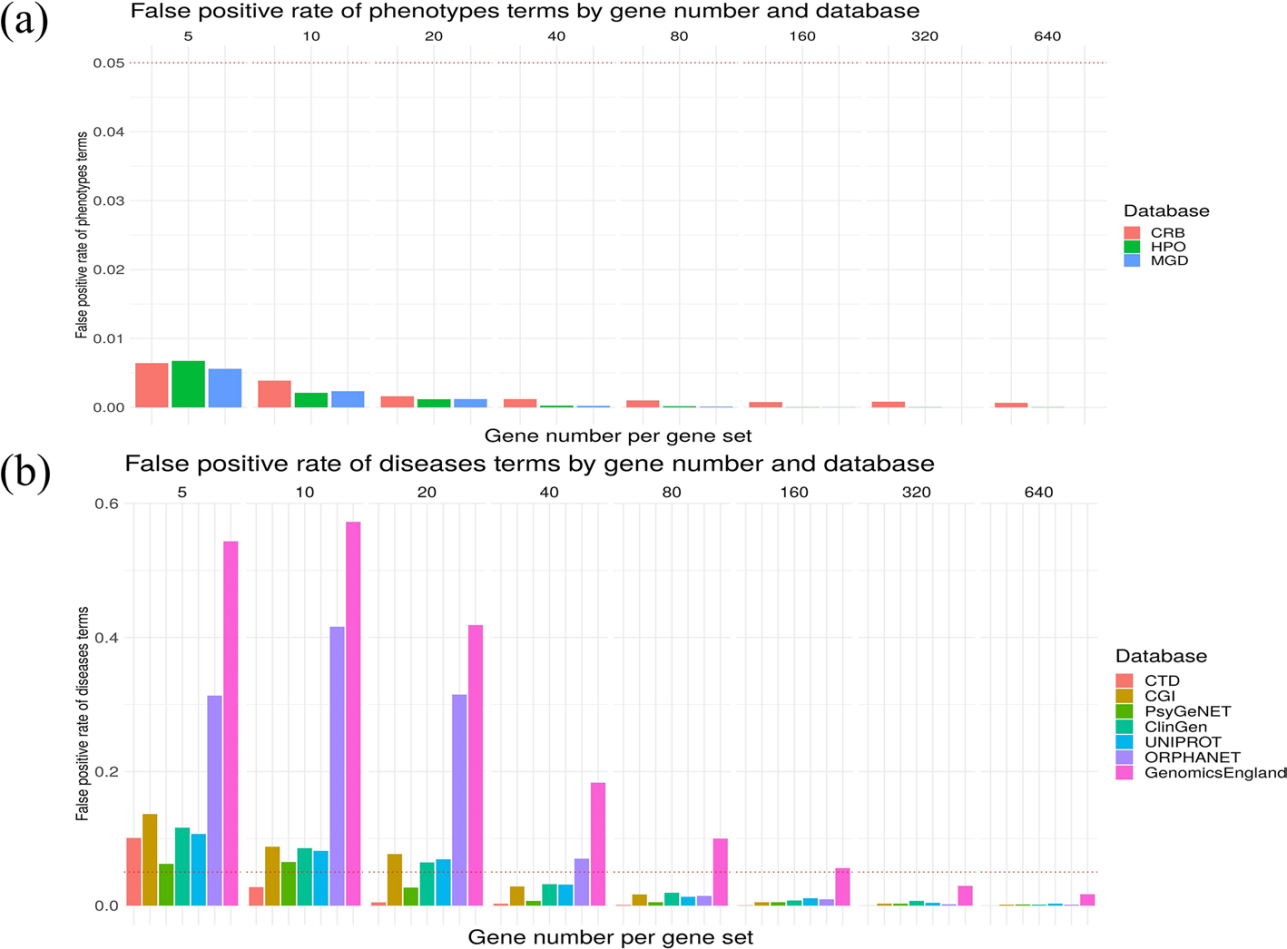
False positive rate of phenotype (**a**) and disease (**b**) terms enrichment across varying gene set sizes (5, 10, 20, 40, 80, 160, 320, 640) per phenotype/disease database. As the simulation points out, CRB, HPO, MGD, are perfectly usable for any gene set size, CTD is recommended for gene set sizes over 10, PsyGeNET for 20, CGI, ClinGen and Uniprot for 40, Orphanet for 80 and GEL for gene set sizes over 180

We assessed PhenoExam for type I error given all phenotype/disease databases considered in the task of phenotypic enrichment analysis of gene sets. Firstly, we evaluated the possibility of finding a phenotypic term erroneously enriched, due to random chance, amongst all the terms at the database, for gene sets of varying sizes. For such purpose we performed simulations of phenotype enrichment analysis for different random gene sets with a variable number of protein coding gene sizes (5, 10, 20, 40, 80, 160, 320, 640) tested in all annotation databases. Each combination of gene set size and database was simulated 1000 times, yielding a total of 80,000 simulations. A graphical representation of the summary of results appears in Fig. 3. PhenoExam maintains type I error under control, see Fig. 3, (a) plot, with a significance level of 0.05 as the number of significant tests is always under 0.05 ratio. We observed a negative correlation between gene set size and proportion of false positive tests, r = −0.453, *P* = 0.026. Type I error is harder to control with Genomics England Panel App (GEL) and Orphanet gene sets. PhenoExam only controls type I error when the gene set size is greater than 80 for Orphanet and 180 for Genomics England. We believe that the difficulties in keeping under control type I error are due to the number of average disease terms associated with each gene, i.e. 4.39 for GEL and 7 for Orphanet when for the rest of the disease databases is, on average, 17.7. Moreover, there is a negative correlation between the number of genes per random gene set and the type I error, r = −0.381, *P* = 0.0038. Therefore, both the number of terms associated with each gene and the size of the gene sets used as input are crucial to obtain enough gene-phenotype relationships to maintain in this way, type I error under control. For these reasons, we recommend using CTD, HPO, MGD or CRB for analyses implying gene sets of size 10. These are, roughly, less than the number of genes we can find in many biological pathway. We recommend using PsyGeNET, ClinGen, UNIPROT or CGI with 40 genes or more. These usually are less than the number of genes detected at most genome-wide association studies. We only recommend the inclusion of the Orphanet and GEL when we have at least 80 and 180 genes respectively. Users can find more information about what database they need to use at https://alejandrocisterna.shinyapps.io/phenoexamweb/#section-help

### PhenoExam differentiates between gene sets with very similar phenotypes

**Fig. 4:**
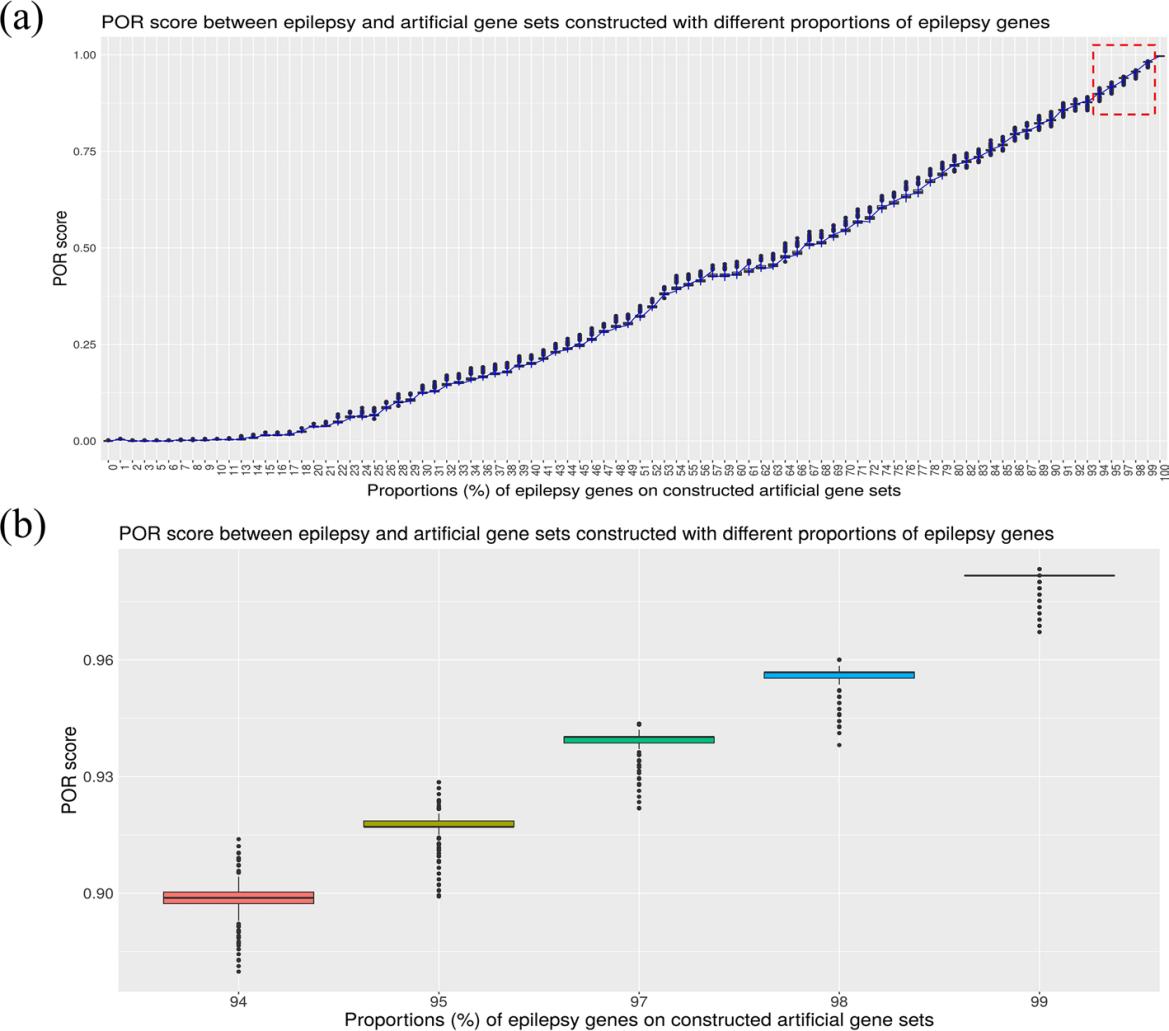
POR significance test between the real and the artificial gene sets constructed with different proportions of epilepsy genes (**a**) and detailed zoom of POR score between the real and the artificial gene sets constructed with different proportions of epilepsy genes (94–99% epilepsy genes). **a** We observed a positive linear relationship between POR and the proportions of epilepsy genes in the artificial gene sets, 0.9674 R^2^ (*P* < 2.2 × 10^−16^). **b** PhenoExam can distinguish well amongst the epilepsy real gene set and the artificial gene sets constructed with high proportions of epilepsy genes (94–99% epilepsy genes) that gather very similar phenotypes with a t-test in all cases (*P* < 2.2 × 10^−16^)

We evaluated how accurate PhenoExam is when computing the POR (detecting phenotype similarities) between gene sets by comparing genetic forms of epilepsy (261 genes from NIMGenetics epilepsy panel) and “artificial” gene sets constructed with variable POR with the original epilepsy gene set and additional genes with similar phenotypic connectivity not associated to epilepsy. In these additional genes we injected a 5% of noise with genes associated with epilepsy phenotypic terms. We performed 1000 simulations for the artificial genes sets (261 genes) constructed with different proportions of epilepsy genes between (0–100%) and different proportions of other genes (0–100%). We calculated the POR significance test between the real and the artificial gene sets (Fig. 4). PhenoExam is sensitive in detecting differences between gene composition changes (≅ 1%) in different gene sets, which in this case are 3 genes. We observed a positive linear relationship between POR and the proportions of epilepsy genes in the artificial gene sets, 0.9674 R^2^ (*P* < 2.2 × 10^−16^) (Fig. 4a). We assessed that PhenoExam can distinguish well amongst the epilepsy real genes and the artificial gene sets constructed with high proportions of epilepsy genes (94–99% epilepsy genes) that gather very similar phenotypes with a t-test in all cases (*P* < 2.2 × 10^−16^) (Fig. 4b).

### Case 1: The analysis between juvenile-onset Parkinson’s disease (PD) and early onset dystonia (EOD) reveals they hold phenotype-level similarities but also potentially interesting differential phenotypes

**Fig. 5:**
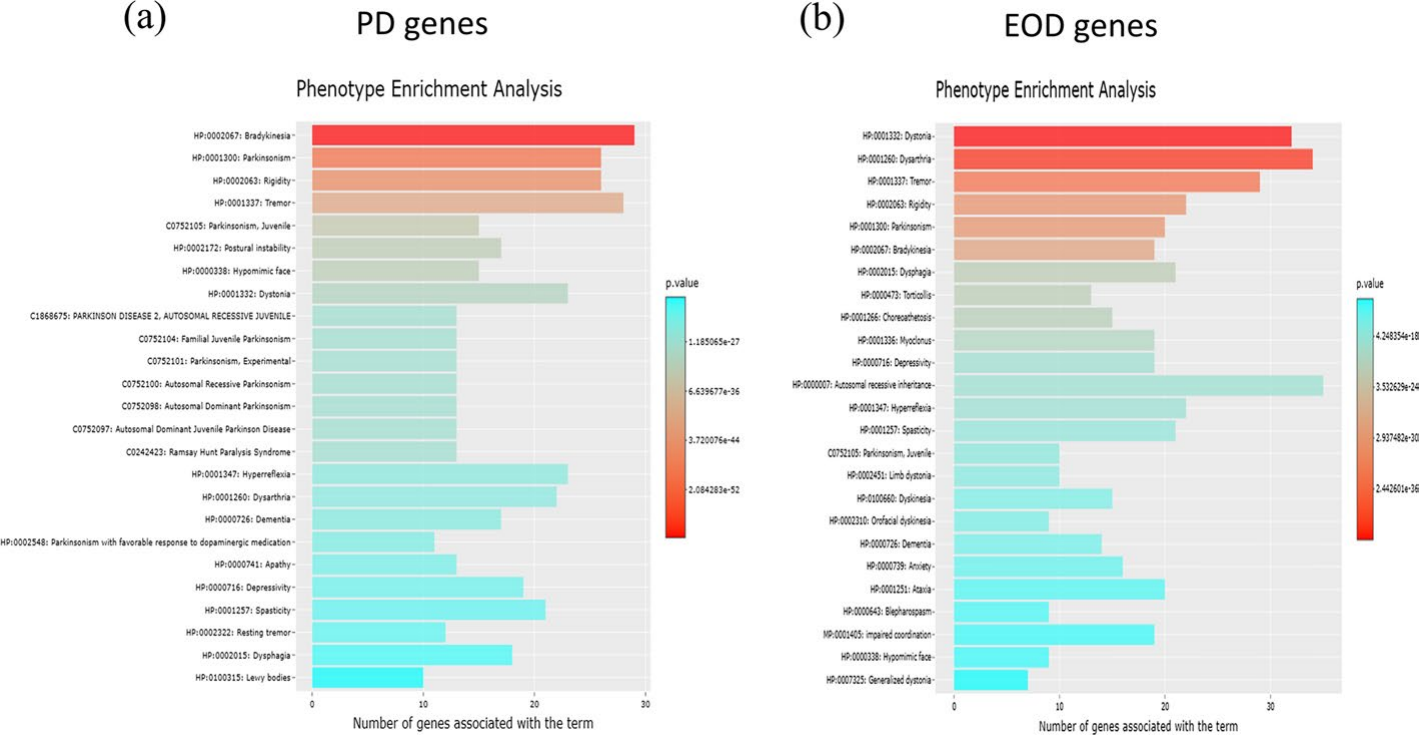
Phenotype Enrichment Analysis in PhenoExam for each gene set. The graph shows the 25 most enriched terms for PD genes (**a**) and for EOD genes (**b**)

We applied PhenoExam to the detection of differential phenotypes between gene sets by comparing two genetic diseases with similar symptoms: juvenile-onset Parkinson’s disease (PD) and early-onset dystonia (EOD). PD and EOD both are movement disorders, PD is caused by a degeneration in the basal ganglia, and it has predominant symptoms consisting of tremor, rigidity, bradykinesia, postural instability and progressive dementia [40]. EOD is a disease characterized by involuntary muscle contractions leading to abnormal posturing and movements and postures, occurring with or without other neurological symptoms [41]. In our case we compared 35 PD genes and 50 EOD genes from Genomics England PanelApp (Additional file 1), with 19 genes in the overlapping set (54.3% of genes on PD gene set). We ran a separate phenotype enrichment analysis for PD and EOD, using HPO, MGD, CTD and CRISPRBrain databases simultaneously (given the simulation analyses performed above, these are the databases recommended by PhenoExam) (Fig. 5). We obtained a table for PD (Additional file 2: Table S1) and EOD (Additional file 3: Table S2). The top two most enriched phenotypes, in each input database, for PD genes were Bradykinesia (HP: 0002067; *P* = 2.16 × 10^−60^) and Parkinsonism (HP: 0001300; *P* = 2.62 × 10^−51^) for HPO, Abnormal gait (MP: 0001406; *P* = 3.78 × 10^−13^) and Neuron degeneration (MP: 0003224; *P* = 9.98 × 10^−13^) for MGD, Parkinsonism, Juvenile (C0752105; *P* = 7.49 × 10^−28^) and Ramsay Hunt Paralysis Syndrome (C0242423; *P* = 7.49 × 10^−28^) for CTD, and no enrichment found for CRISPRBrain. All the enrichment terms found are supported by the literature [42–45]. At the EOD analysis, we found Dystonia (HP: 0001332; *P* = 3.51 × 10^−42^) and Dysarthria (HP: 0001260; *P* = 5.38 × 10^−41^) for HPO, impaired coordination (MP: 0001405; *P* = 7.4 × 10^−14^) and Abnormal gait (MP: 0001406; *P* = 3.17 × 10^−10^) for MGD, Parkinsonism, Juvenile (C0752105; *P* = 7.4 × 10^−13^) and Ramsay Hunt Paralysis Syndrome (C0242423; *P* = 7.4 × 10^−13^) for CTD, and again no enriched term for CRISPRBrain. Above mentioned phenotype terms are associated with dystonia according to several articles [46–50].

**Fig. 6:**
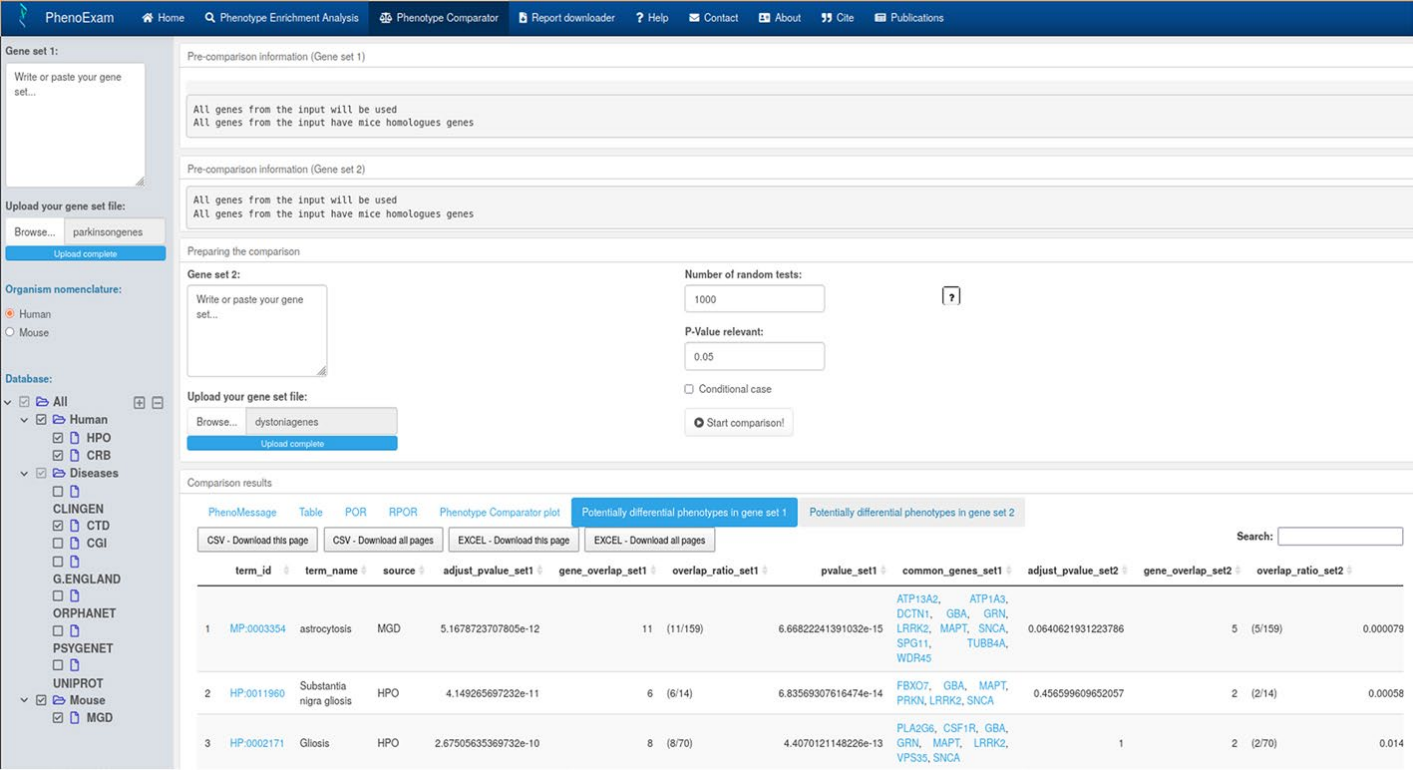
Phenotype Comparator analysis view. We selected PD genes as gene set 1, EOD genes as gene set 2, HPO, MGD, CRISPRBrain and CTD databases and 1000 random tests. We obtained as output interactive tables with the shared phenotypes and the differential phenotypes, plots, PhenoExam phenotype similarities scores and information

We wanted to compare PD and EOD gene sets, through the Phenotype Comparator analysis in PhenoExamWeb (see Fig. 6) using HPO, MGD, CTD and CRISPRBrain as the databases selected, and a randomization based on 1000 null tests. This comparison yielded 139 shared significant phenotypic terms (out of 273 unique significant phenotypic terms in both, POR = 0.509 (*P* < 0.001). Phenotype relevance association analysis for PD and EOD (i.e., whether the shared phenotypes are similar in relevance, i.e., in the number of genes associated with them, within each gene set) results in an adjusted R squared of 0.643 (*P* < 9.23 × 10^−63^) which suggests that an important portion of the common phenotypes are similar in relevance. We actually see they share phenotypic terms such as Tremor (HP: 0001337), Bradykinesia (HP: 0002067), Rigidity (HP: 0002063), Dystonia (HP: 0001332), Abnormal gait (MP: 0001406) or Neuron degeneration (MP: 0003224) (Additional file 4: Table S3). But we also detect differential phenotypes that can be displayed by interactive graphs and tables on the web. For example, significant terms exclusive from the PD gene set phenotypes include Astrocytosis (MP: 0003354; *P* < 5.17 × 10^−12^), Substantia nigra gliosis (HP: 0011960; *P* < 4.15 × 10^−11^), Neuronal loss in central nervous system (HP: 0002529; *P* < 3.74 × 10^−6^), Orthostatic hypotension due to autonomic dysfunction (HP: 0004926; *P* < 9.96 × 10^−6^) and Lewy Body Disease (C0752347; *P* < 1.11 × 10^−3^) (Additional file 5: Table S4). Above mentioned phenotype terms are associated more or only with PD according to several articles [51–56]. The same analysis identified Writer's cramp (HP: 0002356; *P* < 1.37 × 10^−9^) as exclusive to EOD and this refers to a type of focal dystonia [57]. We also found Hypoplasia of the corpus callosum (HP: 0002079; *P* < 3.56 × 10^−5^), a controversial and not widely studied phenotype in dystonia [58, 59] and Acanthocytosis (HP: 0001927; *P* < 2.76 × 10^−3^) a term normally associated with chorea‐acanthocytosis, other disease with dystonia’s similar symptoms [60]. Microcephaly (HP: 0000252; *P* < 4.17 × 10^−4^) is associated with dystonia and several genes such as KMT2B [61, 62]. We also found Intellectual disability, mild (HP: 0001256; *P* < 4.68 × 10^−3^), Dystonia, Primary (C0752203; *P* < 3.26 × 10^−7^) and Hyperactive deep tendon reflexes (HP: 0006801; *P* < 4.31 × 10^−2^) that is associated with Paroxysmal dyskinesia (PxD) [63] (Additional file 6: Table S5).

### Case 2: New likely epilepsy genes predicted by G2PML recapitulate phenotype terms of known epilepsy genes

Let us suppose it is possible to discover new Mendelian genes associated with a specific disease (congenital epilepsy in this case) by finding non-linear patterns of the genes in that panel based on their description through properties based on genomic, transcriptomic and genetics of each gene with machine learning techniques. Therefore, in order to discover new genes, we aim at finding very similar genes in terms of those properties (see G2PML paper at biorxiv [64]). The question we face is: do those genes predicted to be linked to congenital genetic forms of epilepsy recapitulate similar phenotypes to the genes in the panel of origin? The more supportive the answer points to a phenotype recapitulation, the better the predictions made by G2PML. This is an example of what we call a conditional case, comparing phenotypes in gene sets G and G′ when they are disjunct and G′ was generated using G as seeds. More specifically, G refers to epilepsy genes from an in-house maintained epilepsy panel (261 genes) at NIMGenetics. Moreover, G′ is a set of 209 new genes as predicted by G2PML.

We carried out the Phenotype Comparator analysis in PhenoExamWeb with the conditional case option marked, gene set 1 was the epilepsy genes, gene set 2 was the new likely epilepsy genes predicted by G2PML, HPO, MGD, CRISPRBrain and CTD databases selected at the same time and we chose 1000 random tests. We obtained the Pheno Message from PhenoExamWeb that they shared 106 significant phenotypic terms (out of 734 unique significant phenotypic terms in both), which yields a POR of 0.144 (*P* < 0.001). Phenotype relevance association analysis for epilepsy associated genes and epilepsy predicted genes (i.e., whether the shared phenotypes are similar in relevance, i.e., in the number of genes associated with them, within each gene set) results in an adjusted R squared of 0.331 (*P* < 4.35 × 10^−66^) which suggests that an important portion of the common phenotypes are similar in relevance. The *P* values were obtained through the randomization of 1000 random gene sets. We also obtained a table with the phenotypes shared between gene sets (Additional file 7: Table S6). New likely epilepsy genes predicted by G2PML, e.g., DDX3X, KCNH1, TBL1XR1, DLG4 or PDE2A, recapitulate phenotype terms of known epilepsy genes, we check they share epilepsy significant phenotypic terms such as Seizures (HP: 0001250), Global developmental delay (HP: 0001263), Microcephaly (HP: 0000252), abnormal brain morphology (MP: 0002152), hyperactivity (MP: 0001399) and diseases terms without Bonferroni adjust Epilepsy (C0014544) and Autistic Disorder (C0004352). We also found they recapitulate interesting CRISPRBrain terms such as Association with Labile Iron (FeRhoNox Intensity) in Glutamatergic Neuron (CRB: 0000004) and Positive hit with Peroxidized Lipids (Liperfluo Intensity) in Glutamatergic Neuron (CRB: 0000008). Above mentioned phenotype terms are associated with epilepsy according to several articles [65–73]. We also provided the number of genetic variants from the Epi25 whole-exome sequencing (WES) case–control study of each epilepsy gene predicted, we obtained 665 genetic variants in cases and 446 in controls (OR = 1.49) (Additional file 8: Table S7) [74].

## Conclusions

We developed PhenoExam, a freely available R package and Web application, which performs phenotype enrichment and disease enrichment analysis on gene set G, measures statistically significant phenotype similarities between pairs of gene sets G and G′ and detects statistically significant exclusive phenotypes or disease terms, across different databases. PhenoExam just required the names of genes in the gene sets as input and which databases to test for enrichment. It allows us to switch from the gene space and the phenotype space. PhenoExam integrates phenotype data from different databases. And each database is focused on specific diseases and organisms. Therefore, choosing a database for the analyses requires of a basic knowledge of the user about the diseases used there to appropriately understand the analysis outcome. PhenoExam can identify the statistically significant and differential phenotypes of a gene set as we showed with PD, EOD, epilepsy, and likely epilepsy predicted genes. We proved with simulations that it is useful to distinguish between gene sets or diseases with very similar phenotypes through projecting genes into their annotation based phenotypical spaces. With the PD and EOD example above, we clearly see they hold phenotype-level similarities but also potentially interesting differential phenotypes. The conditional case studied between epilepsy associated and epilepsy predicted genes show they hold epilepsy phenotype terms in common, which is useful for the validation of computationally epilepsy predicted disease genes. Therefore, PhenoExam effectively discovers links between phenotypic terms across annotation databases by integrating different annotation databases. All these findings are supported with interactive plots (see tutorials at GitHub project) to foster the visualization and interpretation of findings.

### Availability and requirements

Project name: PhenoExam.

Project home page: https://alejandrocisterna.shinyapps.io/phenoexamweb/

Source code is available at https://github.com/alexcis95/PhenoExam

Operating system(s): Windows, Linux, Mac OS.

Programming language: R language.

License: GPL-2|GPL-3 Any restrictions to use by non-academics: none.

## Supplementary Information

**Additional file 1**. PD and EOD genes. PD and EOD genes selected from Genomics England PanelApp.

**Additional file 2: Table S1.** PhenoExam enrichment analysis using PD genes. PhenoExam output using PD genes.

**Additional file 3: Table S2.** PhenoExam enrichment analysis using EOD genes. PhenoExam output using EOD genes.

**Additional file 4: Table S3.** PhenoExam comparator analyses between PD and EOD genes. Shared phenotypic and diseases terms between PD and EOD genes.

**Additional file 5: Table S4.** Significant terms exclusive from the PD genes. Significant phenotypic terms exclusive from the PD genes and the results obtained in EOD genes.

**Additional file 6: Table S5.** Significant terms exclusive from the EOD genes. Significant phenotypic terms exclusive from the EOD genes and the results obtained in PD genes.

**Additional file 7: Table S6.** PhenoExam comparator analyses between epilepsy and epilepsy predicted genes. Table with data from the phenotypes shared between gene sets.

**Additional file 8: Table S7.** Genetic variants detected from Epi25 whole-exome sequencing in epilepsy predicted genes. Data and number of genetic variants from the Epi25 whole-exome sequencing (WES) case-control study of each epilepsy gene predicted, we obtained 665 genetic variants in cases and 446 in controls.

## Data Availability

Project home page: https://alejandrocisterna.shinyapps.io/phenoexamweb/ Source code is available at https://github.com/alexcis95/PhenoExam Documentation: https://raw.githack.com/alexcis95/PhenoExamWebTutorials/main/tutorial.html Data: HGNC: https://www.genenames.org/download/statistics-and-files/ (protein-coding gene). HPO: https://hpo.jax.org/app/data/annotations (phenotypes_to_genes.txt) https://archive.monarchinitiative.org/latest/tsv/gene_associations/ MGI: http://www.informatics.jax.org/downloads/reports/index.html#go (MGI_PhenoGenoMP.rpt, HMD_HumanPhenotype.rpt, VOC_MammalianPhenotype.rpt). CRISPRBrain: https://crisprbrain.org/simple-screen/ (Exporting each screen name). UniProt: https://ftp.uniprot.org/pub/databases/uniprot/current_release/knowledgebase/complete/docs/humsavar.txt CTD: http://ctdbase.org/downloads/#gd (CTD_genes_diseases.csv.gz). Orphanet: https://github.com/Orphanet/Orphadata_aggregated/tree/master/Genes%20associated%20with%20rare%20diseases ClinGen: https://search.clinicalgenome.org/kb/downloads (Gene-Disease). The Genomics England PanelApp: https://panelapp.genomicsengland.co.uk/panels/ (Each panel). CGI: https://www.cancergenomeinterpreter.org/2018/data/catalog_of_validated_oncogenic_mutations_latest.zip?ts=20180216 PsyGeNET: http://www.psygenet.org/ds/PsyGeNET/results/all_GeneDiseaseAssociations.tar.gz

## Abbreviations

CGI: Cancer Genome Interpreter
ClinGen: Clinical Genome Resource
CTD: Comparative Toxicogenomics Database
EOD: Early-Onset Dystonia
GEL: Genomics England Panel App
HGNC: HUGO Gene Nomenclature Committee
HPO: Human Phenotype Ontology
MGD: Mouse Genome Database
RPOR: Relaxed Phenotypic Overlap Ratio
OMIM: Online Mendelian Inheritance in Man
PD: Parkinson’s disease
POR: Phenotypic Overlap Ratio
